# Knowledge and practice of health workers towards maternal and child health in the Democratic Republic of the Congo: a cross-sectional study

**DOI:** 10.1186/s12978-024-01801-5

**Published:** 2024-05-02

**Authors:** Britou Ndela, Adrien N’siala, Philippe Ngwala, Albert Kalonji, Felix Minuku, Harmonie Bokole, Pascal Kemaina, Jean-Jacques Masumbuku, Ngoma Miezi Kintaudi, Bien-Aimé M. Mandja

**Affiliations:** 1 SANRU Asbl, Kinshasa, Democratic Republic of the Congo; 2 Faculty of Medicine, University of Bandundu, Bandundu, Democratic Republic of the Congo; 3 Association pour l’Education Communautaire, la Préservation de l’Environnement et la Santé (EDEN SANTE Asbl), Kinshasa, Democratic Republic of the Congo

**Keywords:** Knowledge, Practice, Democratic Republic of the Congo, Kasai Province, Maniema Province, Maternal and child health, Health workers

## Abstract

**Background:**

The burden of maternal and child mortality is high in the Democratic Republic of the Congo (DRC). While health workers (HWs) with adequate knowledge and practice of maternal and child health (MCH) are crucial to reduce this burden, the skill level of HWs in charge of MCH in the DRC is currently insufficient. This study aimed to assess the knowledge and practice of HWs towards MCH in Kasai and Maniema, two DRC provinces with very high maternal mortality ratios and under-5 mortality rates.

**Methods:**

This cross-sectional study was conducted in 96 health facilities of Kasai and Maniema provinces in 2019. All HWs in charge of MCH were eligible for the study. Data were collected using a structured questionnaire containing 76 questions on knowledge and practice of MCH. Analyses were performed using the Wilcoxon-Mann-Whitney test, Kendall’s correlation test, and a multivariate linear mixed regression model.

**Results:**

Among participating HWs, 42.6% were A2 nurses (lowest qualification), 81.9% had no up-to-date training in MCH, and 48.4% had only 1-5 years of experience in MCH. In the two provinces combined, about half of HWs had poor knowledge (50.6%) and poor practice (53.3%) of MCH. Knowledge and practice scores were higher in Maniema than in Kasai (*P* < 0.001). Good knowledge and practice scores were significantly associated with high qualification (*P* = 0.001), continuing up-to-date training in MCH (*P* = 0.009), and 6 years of experience or more in MCH (*P* = 0.01).

**Conclusion:**

In Maniema and Kasai provinces, about half of HWs had poor knowledge and poor practice of MCH. The conversion of A1 nurses into midwives as well as the provision of up-to-date training in MCH, supervision, and mentorship could improve the skill level of HWs and could thus reduce the burden of MCH in the DRC.

**Supplementary Information:**

The online version contains supplementary material available at 10.1186/s12978-024-01801-5.

## Plain Language summary

This study assessed the knowledge and practice of health workers (HWs) towards maternal and child health (MCH) in Kasai and Maniema, two provinces of the Democratic Republic of the Congo (DRC) with very high maternal and child mortality rates. About half of surveyed HWs had poor knowledge and poor practice of MCH. Good knowledge and good practice were associated with high qualification, up-to-date training, and 6 years of experience or more in MCH. The conversion of A1 nurses into midwives as well as the provision of up-to-date training in MCH, supervision, and mentorship could improve the skill level of HWs and could thus reduce the burden of MCH in the DRC.

**Supplementary Information:**

The online version contains supplementary material available at 10.1186/s12978-024-01801-5.

## Background

Most low income-countries have unacceptably high maternal and child mortality, with Sub-Saharan Africa and Southern Asia alone accounting for almost 88% of maternal deaths and 80% of under-5 deaths [1–4]. The leading causes of maternal mortality are postpartum hemorrhage, infection (sepsis), hypertension, and unsafe abortion [1, 2]. Neonatal mortality is mainly caused by neonatal asphyxia, prematurity, and infections, while under-5 deaths are mostly due to infections (malaria, pneumonia, and diarrhea) [3, 5].

To reduce the burden of maternal and child mortality, the World Health Organization recommends integrating a package of high-quality maternal and child care in health facilities (HFs) [6, 7]. This care is organized using a number of packages of reproductive, maternal, newborn and child health interventions. Reproductive health is improved by focusing on four main interventions: family planning, those related to adolescent sexual and reproductive health, reduction of unsafe abortion and gender-based violence. Interventions aimed at preventing and treating postpartum hemorrhage, preeclampsia and eclampsia, obstructed labor and maternal sepsis are mainly used to reduce maternal mortality. The stillbirths and newborn mortality can be prevented by the following key interventions: nutritional interventions, antenatal prevention and treatment of maternal infections, intrapartum interventions, newborn resuscitation, postnatal care and management of neonatal sepsis. Vaccination, nutritional interventions infection prevention and treatment are the main interventions for reducing infant mortality [2, 6, 7]. The effective implementation of this package requires sufficient inputs (sustainable infrastructure, essential medicines, material and equipment, and data collection tools) and qualified staff members [6, 7]. In particular, health workers (HWs) with adequate knowledge and practice of maternal and child health (MCH) are needed to ensure the success of interventions [8, 9].

The burden of maternal and child mortality in the Democratic Republic of the Congo (DRC) is among the highest worldwide, with a maternal mortality ratio (MMR) and an under-5 mortality rate (U5MR) estimated at 846 deaths per 100,000 live births and 104 deaths per 100,000 live births, respectively [2, 3, 10]. In 2015, the DRC was far from having achieved the Millennium Development Goals 4 and 5 [5, 11]. As several studies indicate, this dire situation is partly explained by the low skill level of HWs in charge of MCH in the country. Thus, one of these studies was focused on assessment of needs in emergency obstetric care (EmOC) in three provinces (Kinshasa, Bandundu and Bas-Congo) of DRC. This research found that only 3.4 % of health facilities provided EmOC. This low availability and poor quality of EmOC as being due mainly to insufficient number of skilled (more than half have not received a basic competency-based clinical training as a midwife) and trained HWs (only 14% of general practitioners, 9% of midwives and 6% of general nurses have received continuing up-to-date training in EmOC) [12]. The main limitation of this study is that it was carried out only in three provinces in the west of the DRC, and did not cover the provinces in the central and eastern parts of the country, where Kasai and Maniema are located respectively.

Another research, which was covered the whole of the DRC, draw up a mapping intervention in maternal, neonatal and heath. This work showed that the majority of health facilities provided insufficient and poor quality EmOC services. This situation may be partly due to a shortage of up-to-date trained personnel because this study highlighted that less than 3 out of 10 surveyed HFs (28%) had staff up-to-date trained in MCH [13].

The study by Mpunga Mukendi et al. assessed the availability, quality and equity of EmOC in the DRC and was conducted on national scale in this country. This research found that only 9.1% of HF offered basic EmOC and 2.9% of HF offered comprehensive EmOC. The authors identified the insufficient number of skilled and up-to-date trained HWs as one of the reasons for the low availability and quality of EmOC in 1,555 HFs of the DRC [14].

Lastly, the study by Casey et al. was focused on determining availability, utilization and quality of EmOC and FP services. This research was carried out at nine general referral hospitals in five provinces in the DRC (Kasai Occidental, Kinshasa, Maniema, Province orientale and Sud-Kivu). This work showed that none of the hospitals offered quality basic or comprehensive EmOC and this was associated with a shortage of up-to-date trained HWs [15]. Although this study was carried out in the provinces of Kasai Occidental and Maniema, it only involved 2 HFs in the first province and a single HF in the second, making it impossible to assess the overall situation of maternal and child health in these two provinces.

Furthermore, not only (with the exception of the first study) did the above studies fail to assess the skill level of HWs in charge of MCH and to our knowledge, almost no research addressing this problem was conducted in Kasai and Maniema, despite the fact that these two DRC provinces have high MMR and U5MR [10].

This study aimed to assess the knowledge and practice of HWs towards MCH in the DRC provinces of Kasai and Maniema.

Findings of this study would provide the information needed to plan continuing up-to-date training in MCH and to improve the qualification of HWs in order to strengthen maternal child health services delivery in these two provinces. This way, our findings would help policy makers adopt strategies to improve availability and quality of MCH care in these two provinces.

## Methods

### Study design

This cross-sectional study was conducted in 96 HFs of Kasai and Maniema provinces in 2019.

### Setting

Kasai province is located in the south-central region of the DRC and has a total area of 95,631 km^2^. The projected population of the province in 2021 was 5,317,125 inhabitants. Farming, industrial, and artisanal mining are its main economic activities. Kasai is divided into 18 health zones (HZs), each with a GRH. It is made up of 395 health areas [16].

Maniema province is located in the eastern DRC. The province has an area of 132,250 km^2^ and had an estimated population of 2,921,621 in 2021. The main economic activities are subsistence farming, fishing, and artisanal mining. Maniema is also divided into 18 HZs, each with a GRH. It is composed of 279 health areas [17].

In 2019, the MMR was estimated at 131 deaths per 100,000 live births in Kasai and 204 deaths per 100,000 live births in Maniema. The neonatal death rate was estimated at 1.80 per 1,000 live births in Kasai and 5 per 1,000 live births in Maniema. For the same year, the U5MR was estimated at 169 deaths per 100,000 live births in Kasai and 91 deaths per 100,000 live births in Maniema [16, 17].

### Study population and sampling procedures

All HWs in charge of MCH in the 96 HFs of Kasai and Maniema were eligible for the study. However, only midwives and general nurses were included in the sample as these were the main HWs in charge of MCH in the two provinces. Three categories of general nurses were surveyed: A2 nurses, A1 nurses, and L2 nurses. In DRC, midwives have a bachelor’s degree (3 years of university education) and during their course, they are trained exclusively in antenatal, postnatal, and newborn care. They learn how to manage normal deliveries, identify danger signs and refer patients in time. L2 and A1 nurses receive training mainly in general nursing. In addition, they learn certain aspects of MCH care, in particular prenatal care, delivery care, immediate postnatal care and newborn care. L2 nurses receive much more comprehensive training, as they have 5 years of university education while A1 nurses have a bachelor’s degree. As for A2 nurses, they are low-skilled HWs with no university degree (4 years of secondary school and 4 years of nursing school). They receive important information on general nursing and MCH care. All these categories of HWS are assigned to HFS by the provincial human resources office or the first provincial office.

Although the four categories have notable differences in their competency-based training curriculum, we felt it appropriate to include them all in our survey, as they are all currently on the front line in DRC HFs for MCH care. It is therefore important to assess the level of knowledge and practice of all HWs towards MCH in order to help policy makers adopt new strategies to improve the competence and qualification of these health personnel. This approach has also been used in other studies carried out in Africa [8].

General nurses and midwives who were absent on the day of the survey were excluded from the study.

The formula for cross-sectional studies was used to compute sample size [18]. Assuming a proportion of knowledge and practice of 50% (when this proportion was unknown), a confidence level of 95%, a margin of error of 5%, and a non-response rate of 10%, the required sample size was 420 respondents. This number was rounded to 480. The number of respondents per province was proportionate to the population of each province.

Sampling was conducted at two levels. At the first level, all 36 GRHs of Kasai and Maniema were included in the sample. Systematic random sampling was performed among health centers that met the following criteria: (a) a population of more than 10,000 inhabitants; (b) a high MMR (nearly or greater than to 131 deaths per 100,000 live births in Kasai and to 204 deaths per 100,000 live births in Maniema, which corresponded to the average MMR value for each province) ; (c) a high U5MR (nearly or greater than to 169 deaths per 100,000 live births in Kasai and to 91 deaths per 100,000 live births in Maniema, which corresponded to the average U5MR value for each province) ; and (d) a high rate of postpartum obstetric complications (greater or equal to 20%). A total of 31 health centers from Kasai and 29 health centers from Maniema were selected proportionate to the number of health centers in each province. Overall, 96 HFs were included in the sample. Nearly 90% and 85% of the population in surveyed HFs had geographical access to maternal and child health service respectively in Kasai and in Maniema [16, 17].

At the second level, 5 HWs per GRH were randomly selected to meet the required sample size. All HWs working in the selected health centers were included in the sample as all of them were in charge of MCH.

### Data collection procedures

Data were collected using a structured questionnaire (Additional file 1) The questionnaire collected information on the skill level of respondents and included 76 questions covering two dimensions of MCH: knowledge of MCH (34 questions) and practice of MCH (42 questions). Each question could be answered with: “yes,” “no”. Correct answers were given a score of 1 point. Incorrect answers were given a score of zero points. The total knowledge score ranged from 0 to 34 while the total practice score ranged from 0 to 42.

The Cronbach’s alpha of the questionnaire was estimated at 0.75 in a pilot study, showing its reliability [19].

Regarding validity (or psychometrics characteristics) of used questionnaire, although not assessed in our study, it was developed by adapting questionnaires from three other knowledge and practice studies towards MCH [8, 10, 11], which assessed the validity of used questionnaires. Further modification of questionnaire was performed based on current national guidelines. A number of essential questions under each domain of knowledge and practice was included to ensure that the tool contained the most important information for assessing the level of knowledge and practice of HWS towards MCH.

### Dependent variables

Levels of knowledge were measured with 6 composite variables corresponding to different domains of knowledge of antenatal care (ANC), labor care, and immediate neonatal care: key elements of an ANC visit; routine interventions and measurements to be performed during an ANC visit; diagnostic criteria for labor; signs of postpartum hemorrhage; signs of pre-eclampsia; and diagnostic criteria for neonatal asphyxia.

Levels of practice were measured with 6 composite variables corresponding to different domains of practice of ANC, labor care, and immediate neonatal care: management of active management of third stage of labor (AMTSL); management of postpartum hemorrhage; management of pre-eclampsia; management of neonatal asphyxia; management of neonatal infections; and stabilization of low birth weight (LBW) babies.

The 3-level rating scale (from 1 to 100%) developed by Kasanga et al. [9] was used to assess the levels of knowledge and practice of respondents: poor (score below 50%), moderate (score from 51 to 69%), and good (score 70% and above).

### Independent variables

Three first independent variables reflecting the skill level of HWs were considered in the analyses. The qualification of HWs was midwives, L2, A1 and A2 nurses. The continuing up-to-date training in MCH has been estimated by taking into account the number of training sessions received by HWs. The number of years staff has served in MCH care service was considered as third independent variable. Province of work was also included as an independent variable. The total knowledge score and practice score obtained by each HW were also included in the model as independent variables.

### Data analyses

The proportion of correct answers to each of the questions on knowledge and practice of MCH was calculated. As the Kolmogorov-Smirnov test (the *p*-value obtained of 2.2^-16^ provided sufficient evidence of rejection of the null hypothesis)and the Q-Q plot (the quantile points did not lie on the theoretical normal line) showed the distribution to be non-normal, knowledge and practice scores were expressed as medians with their interquartile ranges. The Wilcoxon-Mann-Whitney non-parametric test was used to compare the knowledge and practice scores of HWs between provinces. The relationship between knowledge and practice scores was assessed using Kendall’s non-parametric correlation test. A multivariate linear mixed regression model was performed to determine the relationship between dependent and independent variables. A two-sided alpha level of 0.05 was considered statistically significant.

Data were entered using Microsoft Excel^®^ 2016. Data analysis was performed with R^®^ 4.0.0.

## Results

### Characteristics of respondents

A total of 475 HWs responded to the questionnaire (response rate of 98.9%), including 241 in Kasai and 234 in Maniema.

A2 nurses represented the largest proportion of respondents (42.7%) and L2 nurses the smallest proportion (7.6%). Only 18.1% of respondents were continuously up-to-date trained in MCH. Lastly, 48.4% of respondents had 1-5 years of experience in MCH, while 33.7% had 6-10 years of experience (Table 1).

**Table 1:** Characteristics of respondents

		**Number of respondents (%)**
**Province of work**	Kasai	241 (50.7)
	Maniema	234 (49.3)
**Qualification**	A2	203 (42.7)
	A1	137 (28.8)
	L2	36 (7.6)
	Midwifery	99 (20.8)
**Continuing up-to-date training in MCH**	Yes	86 (18.1)
	No	389 (81.9)
**Years of experience in MCH**	< 1	40 (8.4)
	1-5	230 (48.4)
	6-10	160 (33.7)
	> 10	45 (9.5)

*MCH* Maternal and child health; *A2* 4 years of secondary school and 4 years of nursing school, *A1* bachelor’s degree, *L2* Qualification comprising bachelor’s and honors degree

### Knowledge of MCH

A little over 60% of respondents in both provinces knew the key elements of an ANC visit. Routine interventions and measurements to be performed during an ANC visit were correctly identified by about 50% of respondents in both provinces. Over 60% of respondents in both provinces knew the diagnostic criteria for labor. The signs of postpartum hemorrhage were correctly identified by about 50% of respondents in Maniema and less than 45% of respondents in Kasai. A little less than 50% of respondents in both provinces knew the signs of pre-eclampsia. About 50% of respondents in both provinces knew the diagnostic criteria for neonatal asphyxia (Table 2).

**Table 2:** Correct response rates by domain of knowledge

**Domains of knowledge**	**Maniema (*n*=234) (%)**	**Kasai (*n*=241) (%)**
Key elements of ANC visit	150 (64.1)	149 (62.2)
Routine interventions/measurements to be performed during ANC visit	122 (52.1)	121 (50.2)
Diagnostic criteria for labor	158 (67.9)	155 (64.5)
Signs of postpartum hemorrhage	118 (50.3)	108 (44.9)
Signs of pre-eclampsia	116 (49.6)	115 (47.8)
Diagnostic criteria for neonatal asphyxia	132 (50.4)	117 (48.6)

*ANC* Antenatal care

### Practice of MCH

In both provinces, a little over 50% of respondents knew how to manage AMSTL and about 45% of respondents how to manage postpartum hemorrhage. The steps for managing pre-eclampsia were identified by over 50% of respondents in both provinces. A little over 50% of respondents in Maniema knew how to manage neonatal asphyxia and neonatal infections, while a little less than 50% in Kasai knew how to do so. About 50% of respondents in both provinces knew how to stabilize LBW babies (Table 3).

**Table 3:** Correct response rates by domain of practice

**Domains of practice**	**Maniema (*n*=234) (%)**	**Kasai (*n*=241) (%)**
Management of AMTSL	119 (50.8)	121 (50.1)
Management of postpartum hemorrhage	108 (46.1)	104 (43.2)
Management of pre-eclampsia	122 (52.3)	124 (51.5)
Management of neonatal asphyxia	122 (51.8)	116 (48.3)
Management of neonatal infections	121 (51.6)	115 (47.8)
Stabilization of LBW babies	117 (50.2)	119 (49.4)

*AMTSL* Active management of third stage of labor, *LBW* Low birth weight

### Knowledge scores of respondents

Overall, 50% of respondents had poor knowledge of MCH. The median knowledge score was 20 with an interquartile range of 15 to 22. The knowledge score ranged from 9 to 27. Respondents in Maniema had higher knowledge scores than those in Kasai (*P* = 0.001, Wilcoxon-Mann-Whitney test) (Table 4).

**Table 4:** Knowledge scores of respondents

**Province of work**	**Maximum score**	**Range of score**	**Median [IQR: 1Qu-3Qu]**	**Knowledge score (%)**		
				**poor**	**moderate**	**good**
Maniema	34	11-27	21 [16-22]	50.0	42.4	7.6
Kasai	34	9-24	18 [13-22]	51.7	47.1	3.2
Total	34	9-27	20 [15-22]	50.6	43.8	5.3

*IQR* Interquartile ranges, *1Qu* First quartile, *3Qu* Third quartile

### Practice scores of respondents

Overall, 53% of respondents had poor practice of MCH. The median practice score was 23 with an interquartile range of 20 to 28. The practice score ranged from 12 to 33. Respondents in Maniema had higher practice scores than those in Kasai (*P* = 0.03, Wilcoxon-Mann-Whitney test) (Table 5).

**Table 5:** Practice scores of respondents

**Province of work**	**Maximum score**	**Range of score**	**Median [IQR: 1Qu-3Qu]**	**Practice score (%)**		
				**poor**	**moderate**	**good**
Maniema	42	15-33	26 [21-28]	49.6	45.3	5.1
Kasai	42	12-31	23 [17-28]	57.2	40.7	2.1
Total	42	12-33	23 [20-28]	53.3	43.1	3.5

*IQR* Interquartile ranges, *1Qu* First quartile, *3Qu* Third quartile

### Multivariate linear mixed regression model of the relationship between the skill level of respondents and their knowledge and practice scores

In multivariate linear mixed regression analysis, L2 qualification (*P* = 0.001), midwifery qualification (*P* = 0.001), continuing up-to-date training in MCH (*P* = 0.009), 6-10 years of experience in MCH (*P* = 0.01), and over 10 years of experience in MCH (*P* = 0.01) were significantly associated with good knowledge and practice scores (Tables 6 and 7).

**Table 6:** Multivariate linear mixed regression model of the relationship between the skill level of respondents and their knowledge scores

**Predictor**	**Coefficient**	**SD**	**t**	***p*-value**
**(Intercept)**	0.62	0.36	1.70	0.089
**A2 qualification**	0.05	0.18	0.17	0.646
**L2** **Qualification**	0.83	0.26	3.18	0.001
**Midwifery** **qualification**	0.76	0.24	2.84	0.001
**Continuing up-to-date training in MCH**	0.35	0.16	2.21	0.024
**1-5 years of experience in MCH**	0.15	0.31	0.46	0.639
**6-10 years of experience in MCH**	0.49	0.19	1.98	0.01
**Over 10 years of experience in MCH**	0.51	0.22	2.09	0.01
**Ptotal**	0.75	0.01	51.28	0.0000

*SD* Standard deviation, *A2* 4 years of secondary school and 4 years of nursing school, *L2* Bachelor’s and honors degree, *MCH* Maternal and child health, *Ptotal* Total knowledge score obtained by each HW

**Table 7:** Multivariate linear mixed regression model of the relationship between the skill level of respondents and their practice scores

**Predictor**	**Coefficient**	**SD**	**t**	***p*-value**
**(Intercept)**	2.24	0.43	5.21	0.091
**A2 qualification**	0.04	0.19	0.21	0.836
**L2** **qualification**	0.36	0.21	2.91	0.001
**Midwifery qualification**	0.42	0.18	2.78	0.001
**Continuing up-to-date training in MCH**	0.51	0.19	2.62	0.009
**1-5 years of experience in MCH**	0.36	0.27	1.35	0.176
**6-10 years of experience in MCH**	0.45	0.21	2.30	0.01
**Over 10 years of experience in MCH**	0.40	0.33	2.17	0.01
**Ktotal**	1.12	0.02	51.29	0.0000

*SD* Standard deviation, *A2* 4 years of secondary school and 4 years of nursing school, *L2* Bachelor’s and honors degree, *MCH* Maternal and child health, *Ktotal* Total practice score obtained by each HW

### Correlation between knowledge and practice scores

Kendall’s correlation test showed a strong positive linear correlation between the knowledge and practice scores of respondents (Table 8).

**Table 8:** Correlation between knowledge and practice scores in Kendall’s correlation test

**Kendall’s rank correlation tau**	**z**	***p*-value**
0.9446803	28.321	< 0.001

## Discussion

To our knowledge, this is the first study to evaluate the knowledge and practice of HWs towards MCH in the provinces of Maniema and Kasai in the DRC. A little over half of HWs had poor knowledge (50.6%) and poor practice (53.3%) of MCH in the two provinces combined. However, knowledge and practice scores were higher in Maniema than in Kasai. The multivariate linear mixed regression model found good knowledge and practice scores to be significantly associated with high qualification, continuing up-to-date training in MCH, and 6 years of experience or more in MCH.

Only about half of HWs in Maniema and Kasai knew the routine interventions and measurements to be performed during an ANC visit, a finding similar to those reported in other studies from Sub-Saharan Africa [8, 9]. This situation partly explains current difficulties in preventing common maternal and new-born complications and highlights the importance of improving training in MCH in the DRC [8, 9, 20].

The signs of postpartum hemorrhage, one of the main causes of maternal morbidity and mortality in low-income countries [1, 2, 21], were identified by less than half of HWs in our sample. This level of knowledge is consistent with the findings of other studies from Sub-Saharan Africa [22, 23]. Moreover, in line with what has been observed elsewhere in the region [14, 22], about half of HWs in Maniema and Kasai knew how to manage AMSTL, which is one of the key methods for preventing and managing atonic postpartum hemorrhage [22, 24]. These findings are concerning given that postpartum hemorrhage can cause maternal death within the first two hours if improperly managed [21]. They also suggest that the burden of maternal mortality could be significantly reduced by improving the knowledge and practice of HWs towards this condition.

Only about half of HWs in our study could identify the signs of pre-eclampsia and knew how to manage it. More generally, low levels of knowledge and practice towards hypertensive disease in pregnancy have been reported in several low-income countries [9, 25]. Since eclampsia is an important cause of maternal death in the developing world, it is essential that HWs be properly trained in the management of pre-eclampsia to improve MCH [24, 25].

In our study, only about half of HWs had good knowledge and practice of neonatal asphyxia. Similarly, other studies from Sub-Saharan Africa have shown that a majority of HWs are unable to diagnose and manage this condition [8, 22]. Neonatal asphyxia, which affects nearly 10% of newborns, occurs when HWs fail to perform basic interventions within the first minute after birth to facilitate spontaneous breathing [26, 27]. Accordingly, improving knowledge and practice of neonatal asphyxia would contribute to reducing neonatal mortality.

The key steps of neonatal infection management were identified by less than half of HWs in our sample, a finding that is consistent with other studies from Sub-Saharan Africa [8, 9]. Since infections contribute to two thirds of neonatal mortality in low-income countries [3, 5], ensuring their correct and timely management by well-trained HWs should be a priority.

Lastly, about half of HWs in our study knew how to stabilize LBW babies. This low level of practice is also in line with other studies conducted in the region [8, 9]. Since the stabilization of LBW babies is one of the key methods for preventing hypothermia, infections, and poor breastfeeding [8, 9], the burden of neonatal morbidity and mortality could be significantly reduced by improving HW practice of this technique.

In our study, L2 nurses (*P* = 0.001) and midwives (*P* = 0.001) had higher knowledge and practice scores than A1 and A2 nurses. This finding that can be explained by differences in their competency-based training curriculum. Indeed, L2 nurses in the DRC have a bachelor’s and honors degree (5 years of university education) with more advanced training in MCH care, while midwives have a bachelor’s degree (3 years of university education) with specialized training in antenatal, postnatal, and newborn care. By contrast, A1 nurses have a bachelor’s degree in general nursing with some courses in MCH care. As for A2 nurses, they are low-skilled HWs with no university degree. Our findings suggest that converting A1 nurses into midwives could significantly improve MCH in the DRC.

Unsurprisingly, HWs with 6 years of experience or more in MCH had higher knowledge and practice scores than those with 1 to 5 years of experience (*P* = 0.001).

Moreover, HWs received continuing up-to-date training in MCH had higher scores than other HWs (*P* < 0.001). This finding suggests that MCH could be significantly improved in the DRC through the provision of continuing up-to-date training. Studies conducted in the DRC and Ethiopia have confirmed that a sufficient number of well-trained HWs can play an important role in preventing potentially fatal obstetric complications and, consequently, in reducing the burden of maternal and neonatal mortality [14, 22].

Knowledge (*P* = 0.001) and practice (*P* = 0.03) scores were higher in Maniema than in Kasai, which can be explained by the fact that Maniema has a larger number of L2 nurses and midwives than Kasai.

Lastly, good practice scores were significantly associated with good knowledge scores (*P* < 0.001), confirming that increasing the knowledge of HWs towards MCH can lead to improving their practice.

The main limitation of our study is that data were collected in only 20% of the health centers of Maniema and Kasai. However, the use of systematic random sampling ensured the representativeness of health centers from each province.

Another limitation may be to the fact that we compared the knowledge and practice levels between midwives, L2, A1 and A2 nurses that have different competency-based training. However, all HWs, whatever their level, must provide quality and standard care to women, newborns and children to effectively reduce maternal and child mortality in the DRC.

The last limitation may be related to the fact that we did not evaluate the validity (or psychometrics characteristics) of the questionnaire ourselves. However, our research tool was adapted from other studies, which assessed the validity of used questionnaires.

## Conclusion

In Maniema and Kasai, only about half of HWs had good knowledge and good practice of MCH. These unsatisfactory findings can be explained by the low number of qualified staff and the lack of continuing up-to-date training in MCH in these two provinces of the DRC. The conversion of A1 nurses into midwives as well as the provision of continuing up-to-date training in MCH, supervision, and mentorship could improve the skill level of HWs and could thus reduce the burden of MCH in the DRC.

### Supplementary Information

**Additional file 1.** Questionnaire with 76 questions on knowledge and practice of MCH.

**Additional file 2.** Dataset supporting the conclusions of this article.

## Data Availability

The dataset supporting the conclusions of this article are included in this article and in additional files.

## Abbreviations

AMTSL: Active Management of Third Stage of Labor
A1 nurses: nurses with bachelor’s degree
A2 nurses: nurses with 4 years of secondary school and 4 years of nursing school
ANC: Antenatal Care
DRC: Democratic Republic of the Congo
GRH: General Reference Hospital
HF: Health Facility
HW: Health Worker
HZ: Health Zone
IQR: Interquartile Ranges
LBW: Low Birth Weight
L2 nurses: nurses with bachelor’s and honors degree
MCH: Maternal and Child Health
MMR: Maternal Mortality Ratio
1Qu: First Quartile
3Qu: Third Quartile
SD: Standard Deviation
U5MR: Under-5 Mortality Rate
