# Supplementary material for: Knowledge and practice of health workers towards maternal and child health in the Democratic Republic of the Congo: a cross-sectional study
Source: Reprod Health. 2024 May 2;21:62. doi: 10.1186/s12978-024-01801-5 (PMC11067250; doi:10.1186/s12978-024-01801-5)
Supplement: Supplementary file 1 — Additional file 1. Questionnaire with 76 questions on knowledge and practice of MCH. [file 12978_2024_1801_MOESM1_ESM.docx]

1. **Knowledge**

**I.1. Antenatal care (ANC)**

1. **Key aspects of ANC visit (6 questions)**

Carry out four full visits

Have an individual delivery plan

Health education

Screen for diseases and manage complications

Promote safe childbirth

Promote breastfeeding

1. **Routine interventions and measurements during ANC (11 questions)**

Fetal heart beat

Fundal height

Fetal presentation

Cervical dilation

Descent of the head

Uterine contractions

Maternal blood pressure

Maternal breathing rate

Maternal temperature

Maternal pulse

Maternal urine

**I.2. Labour**

1. **Diagnostic of labour (4 questions)**

Regular uterine contractions

Cervical dilation

Discharge of blood and mucus

Breaking of the waters/ruptured membranes

1. **Signs of hemorrhage (6 questions)**

No contracted uterus

Signs of shock

Amount of external blood

Retained products or retained placenta

Damage to the genital tract

Signs of anemia

1. **Signs of pre-eclampsia (3 questions)**

High blood pressure

Presence of proteins in urine

Edema of face, upper and lower limbs

**I.3. Neonatal care**

1. **Diagnostic of birth asphyxia (4 questions)**

Depressed breathing

Floppiness

Heart rate < 100 beats per minute

Central cyanosis

1. **Practice**
2. **Management of AMTSL (3 questions)**

Immediate oxytocin (1 to 2 min)

Controlled cord traction

Uterine massage

1. **Management of postpartum hemorrhage (8 questions)**

Uterine massage

Give ergometrine or oxytocin IV or IM

Manually remove retained products

Search and repair a damage of the genital tract

Give fluids by IV

Take vital signs

Raise the foot of the bed

Refer to GRH for blood transfusion

1. **Management of pre-eclampsia (4 questions)**

Misoprostol tablet

Diazepam IV

Calcium gluconate IV

Magnesium sulfate IV

1. **Immediate new born care (10 questions)**

Clean the mouth, face and nose

Ensure the baby is breathing

Ensure the baby is dry

Observe for color

Care for the umbilical cord

Provide prophylaxis for eyes

Weigh the baby

Thermal protection (skin to skin)

Begin breastfeeding within first hour

Evaluate/examine baby within first hour

1. **Management of birth asphyxia (7 questions)**

Place new born face up

Wrap baby, except for face and upper chest

Position baby’s head so neck is extended

Aspirate mouth and then nose

Begin ventilation with bag and mask

Stimulate by rubbing back

Explain process to mother

1. **Management of Neonatal Infections (5 questions)**

Continue breastfeeding

Keeping the baby warm

Keep airways clear

Antibiotherapy and consultation

Explain the problem to the mother

1. **Stabilizing the temperature of LBW baby (5 questions)**

Bath baby in water of appropriate temperature

Put on clothes and cover head

Skin to skin contact with mother

Keep in room with temperature of 28-30 degrees Celsius

Near a radiator
